# KIT Mutation and Loss of 14q May Be Sufficient for the Development of Clinically Symptomatic Very Low-Risk GIST

**DOI:** 10.1371/journal.pone.0130149

**Published:** 2015-06-23

**Authors:** Olaf Karl Klinke, Tuba Mizani, Gouri Baldwin, Brigitte Bancel, Mojgan Devouassoux-Shisheboran, Jean-Yves Scoazec, Pierre-Paul Bringuier, Regina Feederle, Anna Jauch, Katrin Hinderhofer, Philippe Taniere, Henri-Jacques Delecluse

**Affiliations:** 1 German Cancer Research Centre (DKFZ) Unit F100, Heidelberg, Germany; 2 Inserm unit U1074, Heidelberg, Germany; 3 Histopathology Cellular Pathology–University Hospitals Birmingham, NHS Foundation, Trust Queen Elizabeth Hospital Birmingham, Mindelsohn Way, Edgbaston Birmingham, B15 2WB, England; 4 Service d’Anatomie et Cytologie pathologiques, Hôpital de la Croix Rousse, 103 Grande-Rue-de-la-Croix-Rousse, Lyon cedex 04, France; 5 Service d’Anatomie Pathologique, Hôpital Édouard-Herriot, 5, place d’Arsonval, 69437 Lyon cedex 03, France; 6 Institute of Human Genetics, University Heidelberg, Heidelberg, Germany; Ecole Normale Superieure de Lyon, FRANCE

## Abstract

The aim of this study was to determine the minimal set of genetic alterations required for the development of a very low risk clinically symptomatic gastro-intestinal stromal tumour within the stomach wall. We studied the genome of a very low-risk gastric gastro-intestinal stromal tumour by whole-genome sequencing, comparative genomic hybridisation and methylation profiling. The studied tumour harboured two typical genomic lesions: loss of the long arm of chromosome 14 and an activating mutation in exon 11 of KIT. Besides these genetic lesions, only two point mutations that may affect tumour progression were identified: A frame-shift deletion in RNF146 and a missense mutation in a zinc finger of ZNF407. Whilst the frameshift deletion in RNF146 seemed to be restricted to this particular tumour, a similar yet germline mutation in ZNF407 was found in a panel of 52 gastro-intestinal stromal tumours from different anatomical sites and different categories. Germline polymorphisms in the mitotic checkpoint proteins Aurora kinase A and BUB1 kinase B may have furthered tumour growth. The epigenetic profile of the tumour matches that of other KIT-mutant tumours. We have identified mutations in three genes and loss of the long arm of chromosome 14 as the so far minimal set of genetic abnormalities sufficient for the development of a very low risk clinically symptomatic gastric stromal tumour.

## Introduction

GISTs are the most frequent mesenchymal tumours of the gastrointestinal tract. They are thought to develop from Cajal cells of the myenteric plexus [1, 2]. The majority of GISTs carry an activating mutation in either the mast cell growth factor receptor *KIT* or the platelet-derived growth factor receptor *α* (*PDGFRA*) tyrosine kinases; an event that is considered as crucial for tumourigenesis [1]. (We shall refer to this kind of GIST as *kinase mutant* henceforth.) Indeed, transgenic mice that carry similar *KIT* mutations develop multiple GIST along the GI tract, although they more frequently succumb of ileus caused by a massive hyperplasia of Cajal cells rather than from neoplastic infiltration [3]. *KIT* mutations found in GIST most frequently occur in exon 11 encoding the kinase’s juxtamembrane domain [1] and lock the protein in its active state. Within exon 11, codons 558, 557, 559 and 560 are affected most frequently, in descending order [4]. For codon 559, Val to Asp (V559D) substitutions dominate Val to Ala (V559A) substitutions. However, the clinical behaviour of GIST with identical *KIT* mutations can widely vary from clinically silent small tumours incidentally discovered at autopsy in elderly patients to locally invasive tumours, some of which metastasise, in particular to the liver [5, 6]. This suggests that additional genetic events condition the variable behaviour of these tumours. Among the so-called *wild-type* GISTs devoid of *KIT* or *PDGFRA* mutations (approximately 10% of all cases), several examples carrying a loss-of function mutation in the Krebs cycle component succinate dehydrogenase (*SDH*) subunits A [7], B or C [8] have been reported. *SDH*-deficient GISTs are known to have DNA methylation profile that is vastly different from that of the tyrosine kinase mutants [9]. Moreover, hypermethylation of certain tumour suppressors such as *RASSF1A* was demonstrated to correlate with aggressiveness and invasiveness [10]. Damaging germline mutations in *SDH* are observed in Carney-Stratakis syndrome, which pre-disposes individuals to developing paraganglioma and GIST [8]. A relatively common event during tumour progression is aneuploidy, in particular loss of chromosome 14q. Up to two out of three GIST suffer partial or complete loss of chromosome 14 [1]. Compared to GIST with other common chromosomal lesions, loss of chromosome 14q and/or 22q was found in a subgroup of GIST with a better prognosis [11] whereas loss of chromosome 1p correlates with more aggressive GIST [12]. Loss of chromosome 14q has been observed in other cancer types and some reports associate this lesion with poorer prognosis. The lesion was reported in ovarian cancer [13] as second most frequent LOH lesion after LOH of chromosome 17, in 40% of a panel of neuroblastomas [14] and 53% of a panel of colorectal carcinoma [15]. Partial loss of 14q seems to distinguish malignant from benign meningioma [16]. Renal cell carcinomas where the von Hippel-Lindau tumour suppressor gene is deleted are more aggressive if in addition 14q is lost [17]. In patients with non-metastatic clear-cell renal cell carcinoma 14q loss is associated with decreased survival [18].

We sought to study the minimal set of genomic and epigenomic alterations that can lead to the occurrence of GIST. Since high-risk tumours are likely to harbour many genomic alterations that are consequences, and not the cause, of tumourigenesis and thereby mask the interesting data in the analysis, we selected a very low-risk clinically symptomatic kinase-mutant gastric GIST. This distinguishes this tumour from so-called mini-GIST that are typically incidentally discovered in resection samples. Here we present the results of a whole genome sequencing analysis as well as of a methylation profiling of this neoplasm.

## Materials and Methods

### Tumours and controls

Fifty-two GIST cases for which fresh frozen tumour tissue was available were included in this study. Samples were retrieved from the files of three Pathology departments located in the UK and France (Queen Elizabeth Hospital Birmingham, Hôpital de la Croix Rousse, Lyon, France and Hôpital Edouard Herriot, Lyon, France). The panel comprised 9 very low-risk, 12 low-risk, 8 moderate and 23 high-risk primary GISTs according to the European Society for Molecular Oncology (ESMO) consensus guidelines [19].

### Ethics statement

Some samples were collected through the University of Birmingham’s Human Biomaterials Resource Centre HBRC that is ethically approved (North West 5 Research Ethics Committee, Haydock Park; Ref 09/H1010/75). Retrieval was approved by the HBRC ethics board (ref. 11-048). The remaining samples were obtained though ethically approved biobanks from the Hospices Civils de Lyon, France (tumorothèque HCL-Croix Rousse and tumorothèque HCL-Edouard Herriot). Both biobanks’ ethics boards approved this study.

### DNA extraction

All tumours and corresponding normal tissues were homogenized and subsequently mixed with lysis buffer (700 μl/g tissue) containing 2.1% (v/v) Proteinase K (10 mg/ml) and 0.7% SDS in TE (pH 8.0). Samples were incubated o/n at 37°C. Phenol extraction followed by butanol treatment and ethanol precipitation was performed as described by Sambrook and Russell [20].

### PCR

DNA fragments of ZNF407 and RNF146 were amplified using 30 ng genomic DNA as a template and the primers listed below. Due to its size, we covered the first exon of ZNF407 by four separately amplified regions, named A, B, C and D. ZNF407 (Exon 1 A):

- 5’-AAAGGGTGTTTCATTGGGGC-3’
- 5’-CACGCTTCCAGGTGTTTCAGC-3’

ZNF407 (Exon 1 B):

- 5’-CTTCTTGTTGAAATGATGCCTTCC-3’
- 5’-TGATAGTCTTGACCATGCCGAAG-3’

ZNF407 (Exon 1 C):

- 5’-GGAAAAGCATCTCAGGAAGAACC-3’
- 5’-TTTGTTGATGCTCTTCTTCAGGC-3’

ZNF407 (Exon 1 D:)

- 5’-CTTCAGAGCCAGAGGACTTCG-3’
- 5’-ATGAGCAGCCACAGGTAGGG-3’

RNF146 (Exon 8):

- 5’-GGTGGTTAGTGTTTTCATAATTG-3’
- 5’-CACCCCAACTCCCAAAATGC-3’

PCR was conducted over 38 cycles according to the manufacturer’s protocol using 0.125 μl Thermo-Start Taq DNA polymerase (5 U/μl; Thermo Scientific) and 20 μmol forward and reverse primer per reaction.

### Copy number SNP array analysis

Molecular karyotyping using 50 ng of tumour DNA was performed with the Affymetrix CytoScan HD Array (Affymetrix, Santa Clara, CA) according to the manufacturer’s recommendations. Bioinformatics was done with the Affymetrix Chromosome Analysis Suite 2.0 software using annotation files version GRCh37/hg19. For analysis and interpretation copy number changes ≥ 100 kb were considered. For the detection of uniparental isodisomies, the sample was checked for regions of homozygosity with a minimal size of 10 Mb. Common CNVs registered in the Database of Genomic Variants (http://projects.tcag.ca/variation) and from the software-internal control cohort were excluded.

### Methylation profiling

Meythlation profiling was performed using the Illumina Infinium HumanMethylation450 BeadChip. DNA was extracted as described above and 500 ng (37.3 ng/μl) processed according to the manufacturers protocol. The raw intensity values (two channels for each probe) were extracted from the Illumina files using the LUMI software package for R [21]. It is known that the two different probe chemistries used in the assay give different distributions of signals. In addition, we noticed that the dynamic range of intensities varies from probe to probe, but consistently across samples. We used the reference signals generated by Bibikova et. al. [22] available from the Illumina website to normalise each probe read-out to a value between 0 and 1, comparable to the commonly used beta value. More details on data processing, the raw signals as well as the normalised values are available at NCBI in the Gene Expression Omnibus database under accession GSE55521. The same processing was applied to the raw intensity values of the HumanMethylation450 dataset GSE34387. For the clustering we extracted 16100 probes with variance exceeding 0.1 across all samples. Based on these 16100 values, we clustered the samples using Pearson’s correlation as distance between samples and complete linkage as distance between clusters.

### Whole genome sequencing

A DNA library generated from 3 μg of DNA from a very low-risk GIST sample was sequenced using 14 lanes of an Illumina HiSeq 2000 machine at the genomics and proteomics core facility at DKFZ. Variants were called using a pipeline developed in the theoretical bioinformatics group at DKFZ as previously described [23]. The pipeline uses samtools mpileup 0.1.17 and bcftools at its core and compares mutations against dbSNP build 135. Additionally, we checked all shown mutations against the latest version of dbSNP available online (www.ncbi.nlm.nih.gov/snp/). The complete lists of single-nucleotide variants and small indels, already classified into silent/coding, germline/somatic (present/absent in the control) etc. were filtered for high-confidence calls of coding variants in exons. Mutations supported by reads on one strand only were excluded. In order to have a reliable estimate on allele frequency, we also excluded regions with sequencing depth of less than 10.

Founding mutations (see results below) among the somatic mutations were searched for as follows. We modelled sequencing of alleles from the impure tumour sample as a Bernoulli process with success probability $\frac{p}{2}$ and *n* trials where *p* is the tumour purity (half the purity for heterozygosity) and *n* is the observed coverage at any given position. A threshold *k* was set to be the largest number where the probability *P*(*X* > *k*) exceeded 5%. This threshold is 1 for *n* ≤ 13, 2 for *n* ≥ 14, 4 for *n* ≥ 19, 5 for *n* ≥ 28, 6 for *n* ≥ 33, 7 for *n* ≥ 37 and so forth. If at least *k* of *n* reads carried the observed mutation, the mutation was assumed to be present in the majority of tumour cells. The threshold is designed so that an expected fraction of 95% of all true founding mutations passing the above quality criteria are included.

### Functional assessment of mutations

To assess the impact of point mutations on protein function, we retrieved the PolyPhen2 HDIV scores [24] from dbNSFP 2.0 [25].

## Results

### Pathological characterization of the sequenced tumour

**Fig 1:**
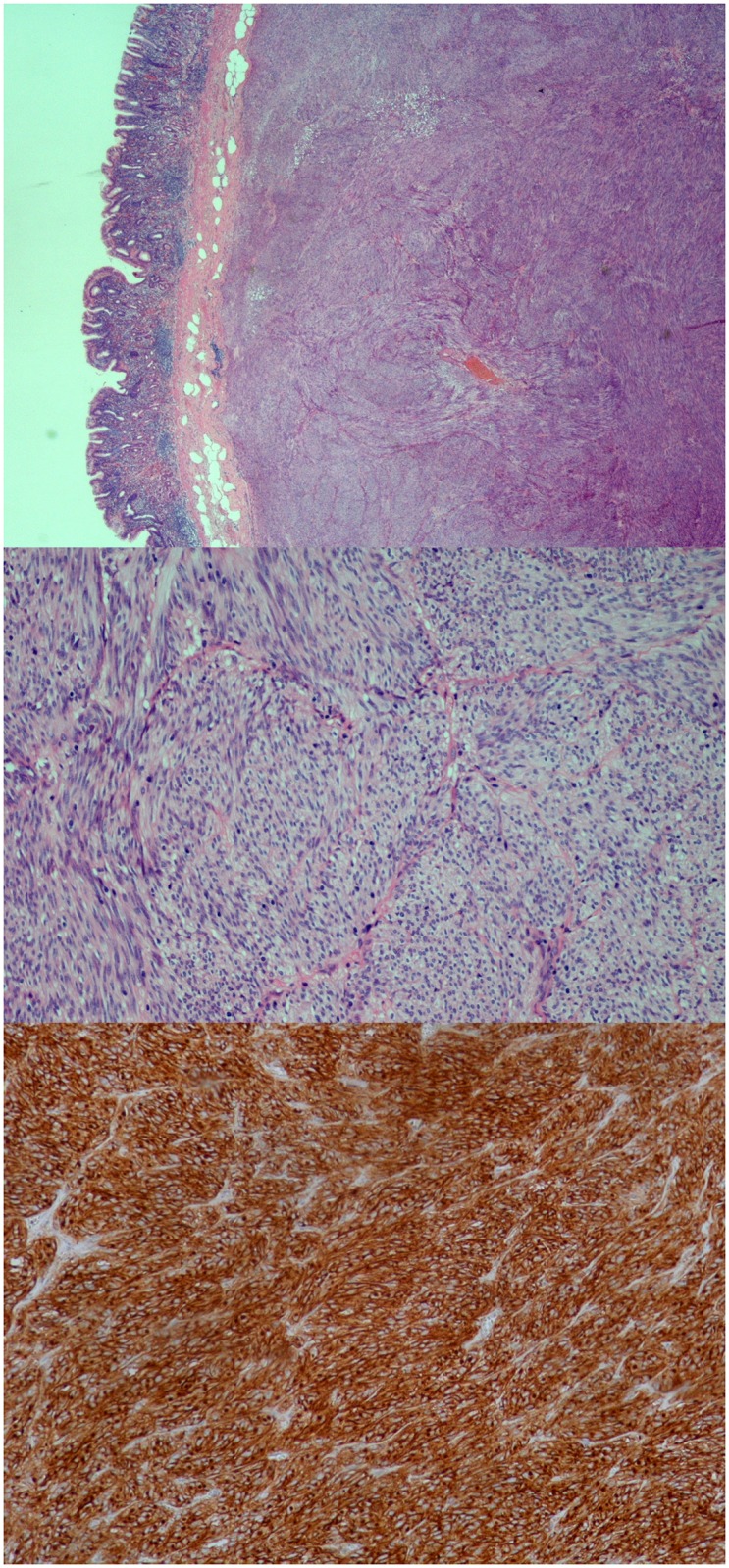
Pathological characteristics of a very low-risk GIST. This H&E staining shows a low (upper picture) and high power view (middle picture) of the GIST that was submitted to whole genome sequencing and how it invades the gut wall. The tumour was also stained with an antibody specific to c-kit (lower picture).

We selected a tumour of the stomach that occurred in a 53-year old patient and measured 3 cm in diameter on the piece of gastric resection. Examination of tissue sections showed that the tumour cells displayed a mitotic rate of 3 per 50 high-power fields. Tumours of this size, location and mitotic rate are classified as very low risk (1.9%) of relapse according to the ESMO guidelines [19] Indeed, the patient was treated by surgical resection only and is alive and free of relapse 5 years post-diagnosis. This GIST had a predominantly spindle cell morphology, expressed CD34, CD117 (KIT), DOG-1 and SDHB (Fig 1 and data not shown).

These proteins were expressed in approximately 60-70% of the cells.

### Genomic characterisation of the sequenced tumour

**Fig 2:**
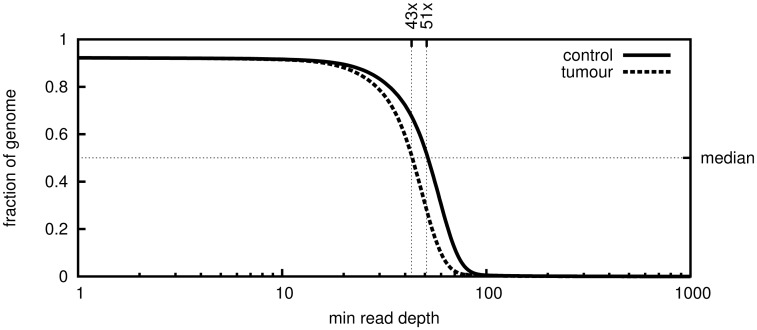
Mapping plot of the sequenced GIST sample. Plotted is the fraction of the genome that is covered to at least a certain depth.

**Fig 3:**
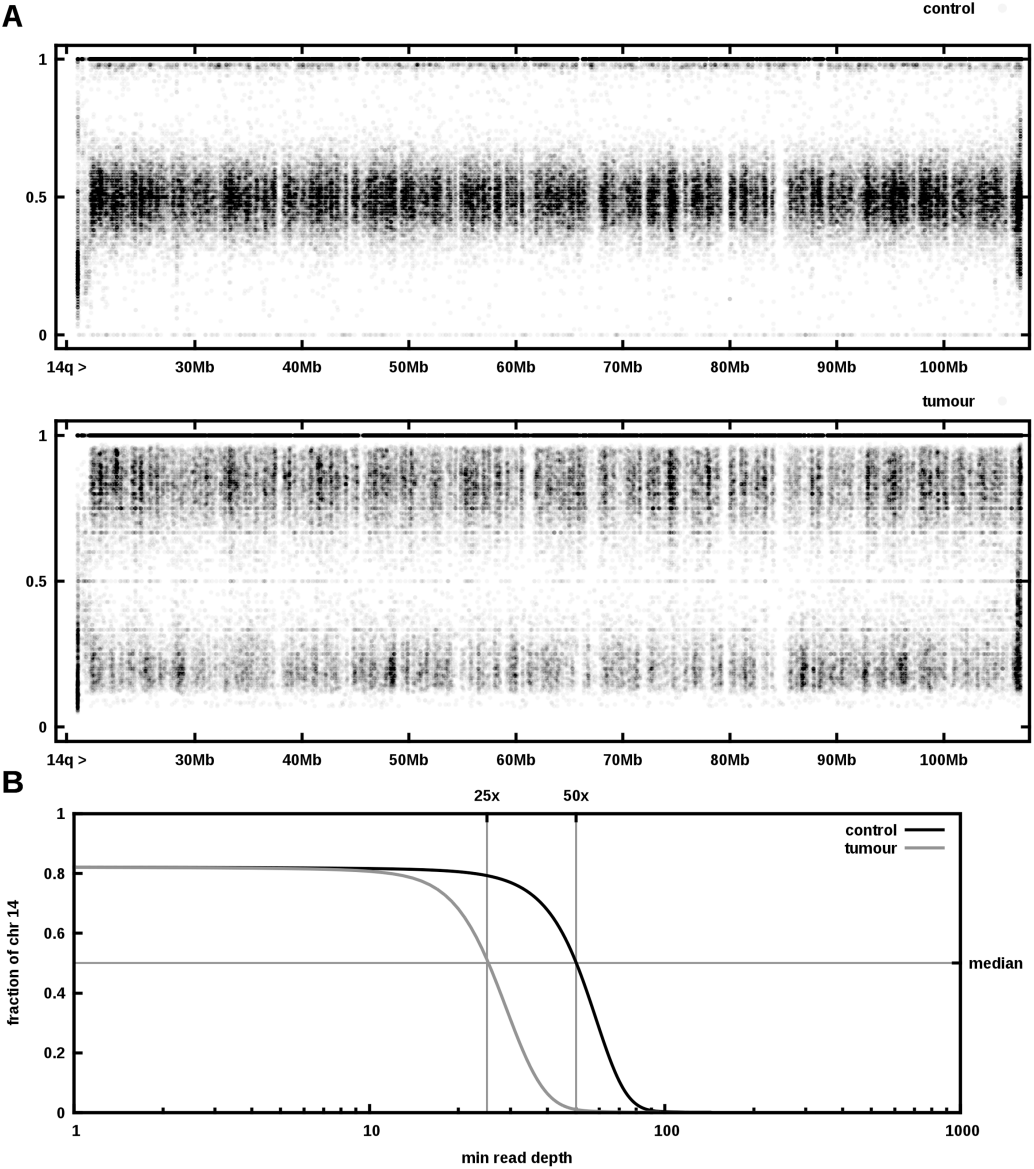
Aneuploidy of the low-grade GIST. *A* Plotted are the allele frequencies of all found single nucleotide polymorphisms along chromosome 14q. While the frequencies in the control (upper panel) are either 1 or approximately 0.5, the tumor sample exhibits frequencies around 0.2 and 0.8. *B* Plotting the read depths for chromosome 14 shows a markedly reduced number of reads for the tumor sample compared to the control.

We extracted DNA from this very low-risk GIST and from healthy control tissue of the adjacent duodenum. Both samples were sequenced and the resulting reads aligned against the human reference genome. About 2.9 × 10^9^ unique reads were aligned against UCSC human reference hg19 using the mapper bwa 0.5.9-r16 resulting in 2.7 × 10^9^ mapped reads, covering the reference by 92% for both control and tumour sample with average depth of 50 and 42 reads, respectively. A depth of at least 30× was achieved on 83% and 79% of the reference, respectively (see Fig 2). Similar to copy number SNP arrays, whole-genome sequencing facilitates detection of copy number variations. We compared the ratio of the number of reads mapping to two alleles of a polymorphic site. Heterozygous variants typically yield a ratio of approximately 1:1 (i.e. allele frequency 0.5) and a shift in this ratio from control to tumour indicates loss or gain of one of the alleles. Since the tumour sample is impure, even complete loss of one allele in the tumour does not mean the observed allele frequency drops to zero. Plotting the observed allele frequencies of identified single-nucleotide polymorphisms (SNPs) clearly shows loss of heterozygosity in the long arm of chromosome 14 (Fig 3A). The median sequencing depth of chromosome 14q in the tumour is precisely half of that in the control (Fig 3B), indicating that one allele was lost. The finding was confirmed by copy number SNP array analysis. The height of bands in the lower panel of Fig 3A matches very well the histologically estimated tumour purity of 60-70%. Besides 14q loss, none of the other chromosomal lesions commonly found in GIST (e.g. loss of 1p or 22q) was detected. Concordant with the immunostaining, whole genome sequencing revealed a heterozygous somatic V559A mutation in exon 11 of *KIT*. Comparing the alignments of the control and tumour sequencing data suggests that, apart from the loss of heterozygosity described above, there are very few genomic differences between tumour and control indeed: A total number of 5509 somatic SNVs and 1784 short insertions/deletions (indels) were found, of which only 173 resp. 17 are located in transcribed regions of the genome. Of these, only 90 SNVs and 1 deletion are non-silent and not previously described polymorphisms.

Whole genome sequencing revealed a germline missense mutation in succinate dehydrogenase subunit B (R90Q, allele frequency 0.5), suggesting that our sample may belong to the class of SDH-deficient GIST. Neither immunostaining (see first paragraph and below) nor its methylation profile, however, supported this hypothesis.

### Methylome of the sequenced tumour

**Fig 4:**
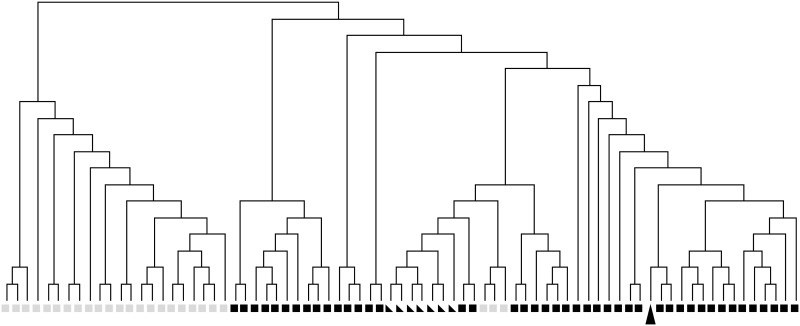
Methylation profile of the low-grade GIST. Shown is a hierarchical clustering of our GIST sample (black arrowhead) and samples in GEO dataset GSE34387 (black squares: SDH positive GIST, grey squares: SDH deficient GIST, triangles: normal tissue)

We probed the methylation levels of ˜450.000 CpG sites using a BeadChip and compared our sample against the analysis published in [9] (GEO dataset GSE34387), where a clear difference between the methylation profiles of SDH-negative and -positive GIST was reported. The clustering displayed in Fig 4 shows that the methylation pattern matches that of a group of SDH positive kinase-mutant GIST and even that of healthy muscular tissue much better than the methylation pattern of most SDH deficient GISTs. Inspection of the probed CpG sites in the promoter and gene body regions of common tumour suppressor genes revealed no significant gene-level deviations from normal tissue (data not shown). Therefore, we can rule out that hyper- or hypomethylation has played a role in formation of the examined low-grade GIST with reasonable certainty.

### Predisposing polymorphisms

We hypothesized that there may be other genomic alterations besides the *KIT* mutation and loss of chromosome 14q that drove the formation of the studied tumour. In particular, we searched for germline polymorphisms known to confer increased risk of developing cancer. Checking all mutations affecting proteins against the latest ClinVar [26] database yielded 48 single-nucleotide variants that have been linked to cancer or other diseases, of which the *KIT* mutation is the only somatic one. We further checked the mutations against the latest version of the *GET-evidence* database (http://evidence.pgp-hms.org/about) of crowd-sourced variant annotations. Among the genes with germline mutations that are possibly pathogenic are *AURKA*, *BUB1B*, *BRCA2*, *MTHFR*, *IRF1*, *TLR4*, *ZEB1*, *LIG4*, *ELAC2*, *FUT3* (all heterozygous) as well as *RET*, *EVC*, *IL4R*, *IL7R* (all homozygous).

### Potential cancer driver mutations

We then turned towards somatic variants and genes commonly associated with GIST. In the genes associated with GIST besides *KIT* and *SDHB*, e.g. *PDGFRA*, *BRAF* [27], *KRAS* [28], *NF1* or *NF2*, only germline polymorphisms that are likely not pathogenic could be found. Therefore we searched for somatic variants affecting genes not commonly associated with this type of cancer. In order to tell founding from passenger mutations, we looked for mutations approximately as frequent in the tumour as the *KIT* mutation. For heterozygous mutations the sampling of alleles in whole-genome sequencing is stochastic, meaning there is always a chance that the unmutated allele is over-represented making the mutation appear not as frequent as it really is. Our inclusion criteria for founding mutations accounts for this (see methods). Twelve non-silent single-nucleotide variants and one frameshift deletion remained.

All sites that were double-checked using Sanger sequencing confirmed the listed mutation (data not shown). While *KIT* mutations are well-known drivers of GIST, tissue-specific genes such as the serine/threonine protein kinase *TESK1*, the myelin regulatory factor *MYRF*, as well as the 5-oxo-L-prolinase *OPLAH* or the zinc influx transporter *SLC39A9* have not been associated with cancer before. Inter-species homology-based methods estimate the amino acid changes caused by these mutations and the ones found in *DOPEY2*, *LAMA5*, *PCDHB11* and *STS* to be likely not damaging for protein function. We could not exclude a priori the possibility that any of the mutations in the relatively uncharacterised genes *RNF146* or *ZNF407* was linked to the formation of the tumor.

To rule out a contribution of *RNF146* or *ZNF407* we screened a panel of 21 frozen tumour samples for the presence of mutations in the fifth exon (in this splice variant) of *RNF146*. However, all these samples were devoid of mutations. We therefore decided to stop the mutation screening for this gene and concentrate instead on the *ZNF407* gene to sequence all its coding exons (altogether 6.8 kb). Screening of 52 GIST tumour samples of various grades uncovered 117 point mutations at 20 different locations, most of which are known polymorphisms (SNPs). In addition to the *ZNF407* mutation listed in Table 1, we found one novel (as of the time of writing) germline mutation. Based on the minor allele frequency of each known polymorphism as estimated from the 1000 Genomes project data, only the first polymorphism in Table 2 was observed a significantly higher number of times than expected (data not shown).

**Table 1:** Potential founding mutations in protein-coding regions.

**Gene**	**Consequence**	**Frequency** ^1^	**dbSNP ID**
KIT^s^	nonsynonymous SNV	14/53	rs121913517
PCDHB11^s^	nonsynonymous SNV	12/32
RNF146^s^	frameshift del	24/52
OPLAH	nonsynonymous SNV	10/29	rs200933200
TESK1^s^	nonsynonymous SNV	11/28
MYRF^s^	nonsynonymous SNV	10/32
SLC39A9	nonsynonymous SNV	24/37
RRN3P1^p^	noncoding variant	8/37
ZNF407^s^	nonsynonymous SNV	17/53	rs368982407
LAMA5^s^	nonsynonymous SNV	10/34
DOPEY2^s^	nonsynonymous SNV	20/59
STS^s^	nonsynonymous SNV	21/25	rs373509256
MAGEB16^p^	nonsynonymous SNV	24/28

^1^, Fraction of sequenced reads showing the mutation; p, pseudogene; s, confirmed by Sanger sequencing.

**Table 2:** Single nucleotide variations in exon 1 of ZNF407 in a panel of 52 GIST samples. The last column shows in how many of the 52 samples the mutation was found.

**Kind**	**dbSNP ID**	**NT**	**AA**	**Pred**	**MAF**	**Samples**
SNP	rs75994611	A181G	Ser61Gly	0	G = 0.0115/24	4
SNP	rs3794942	A206G	Asn69Ser	0.002	G = 0.0744/162	4
SNP	rs200158544	G427T	Val143Phe	0.002	?	1
Novel^a^	–	A898C	Asn300His	0.012	?	1
SNP	rs115368653	G1027A	Glu343Lys	0.005	A = 0.0078/17	1
SNP	rs77148611	A1077G	Val359	–	G = 0.0096/20	2
SNP	rs3794941	T1248C	Pro416	–	C = 0.2020/439	16
SNP	rs17817969	C1263T	Leu421	–	T = 0.079/171	12
SNP	rs191767705	T1521G	Arg507	–	G = 0.0032/7	1
SNP	rs77006793	C1523T	Pro508Leu	0	T = 0.0032/7	1
SNP	rs7227263	G1534A	Gly512Arg	0.001	A = 0.079/171	11
SNP	rs7227391	G1578A	Thr526	–	A = 0.0794/173	12
SNP	rs139800364	T1927C	Leu643	–	C = 0.0014/2	1
SNP	rs374273356	A2174C	Lys725Thr	1.0	?	1
SNP	rs201078153	G2866A	Val956Ile	0.006	?	1
SNP	rs948615	A2915C	Asn972Thr	0.068	C = 0.2025/440	10
SNP	rs73971116	T3059G	Leu1020Trp	0.89	G = 0.0023/4	1
SNP^b^	rs368982407	C3112T	Arg1038Trp	1.0	?	1
SNP	rs12327359	T4507C	Leu1503	–	C = 0.4096/891	35
SNP	rs114095914	T4572G	Thr1524	–	G = 0.0078/17	1

Abbreviations: NT, nucleotide change in NM_017757; AA, amino acid change in NP_060227; Pred, PolyPhen2 HDIV score; MAF, minor allele frequency 1000 Genomes project; a, Germline mutation; b, Somatic mutation.

Interestingly, the somatic mutation in the sequenced GIST as well as two rare polymorphisms in the screened panel are situated within the zinc ion-binding region of one of the protein’s zinc fingers (Table 3).

**Table 3:** Coding mutations in ZNF407 zinc fingers.

AA position	ZNF motif^a^	AA position
	Lys725Thr (rs374273356)	
705	CkkCfyktrsstvltrHi[k/t]lrH	728
	Leu1020Trp (rs73971116)	
1017	C[l/w]hCefsahssaslelHvkrkH	1040
	Arg1038Trp (rs368982407)	
1017	ClhCefsahssaslelHvk[r/w]kH	1040

^a^, The C2H2 zinc finger motif comprises two cysteines and two histidines, shown in capital letters. Positions of amino acid changes are highlighted as [x/x]. Amino acid (AA) positions are internal to protein sequence NP_060227.

## Discussion

The aim of this study was to identify molecular events that are sufficient for the development of a very low risk gastric GIST. We chose a low-risk tumour so that crucial alterations are not drowned out by events that occurred later in tumour progression. Indeed very few somatic mutations were identified that could reasonably have contributed to the formation of this tumour.

There are multiple reports of the particular *KIT* V559A substitution detected in our patient, some in familial GIST [29, 30] as well as for melanomas [31, 32]. In vitro experiments demonstrated that V559A renders the kinase constitutively active [33] and in vivo the responsiveness of V559A-*KIT* to Imatinib treatment was observed [34]. We conclude that the *KIT* V559A mutation contributed to the occurrence of the reported case of GIST.

*RNF146* is expressed in the brain where it protects neurons from programmed cell death [35] which raises the question whether the same holds true for Cajal cells. A recent study [36] correlates *RNF146* overexpression with invasiveness of non-small cell lung cancer. In the light of these findings it is uncertain whether the frameshift deletion we found contributed to tumour formation or rather hindered it. Moreover, it seems that *RNF146* mutations are not commonly associated with GIST since the studied tumour remains the only example among a panel of 21 samples of various GIST types.

*ZNF407* is a member of the large C2H2 (Krüppel) type zinc finger protein subfamily whose members have previously been implicated in cancer development [37, 38]. Depending on the stringency of motif, protein databases predict between 8 and 22 zinc fingers in *ZNF407*. C2H2 zinc fingers contain four zinc-coordinating residues, namely two cysteines (C) and two histidines (H). The amino acids between the last C and the first H determine the DNA sequence recognised by the finger. They are part of an alpha helix which includes the two histidines [39]. Replacing the hydrophilic arginine (R) by the bulky amphipatic tryptophan (W) as in the somatic mutation reported here is likely to inflict damage on the stability of the zinc finger’s alpha helix. Supporting this hypothesis is the low weight of the W two amino acids upstream of the terminal H in the C2H2 motif weight matrix (http://prosite.expasy.org/PS50157), reflecting the fact that W in that position is rarely found in functional zinc fingers. An R to W mutation in a zinc finger’s alpha helix has previously been reported by [38] for the tumour suppressor *CTCF* in Wilms’ tumour. In contrast, the more frequent SNP shown second in Table 3 replaces one hydrophobic amino acid by another such.

A large body of evidence suggests the existence of at least two tumour suppressor genes on the long arm of chromosome 14 [13, 40, 41], although the studies disagree on the exact location. One of the suppressors may be hypoxia-inducible factor 1*α* [18]. After the *KIT* mutation discussed above, the observed loss of the long arm of chromosome 14 is the second genomic abnormality that can with certainty be attributed as causal for tumour development.

Epigenetically, the sequenced tumour seems not to differ significantly from other kinase-mutant GISTs. Comparison with normal tissue is difficult, since GIST derive from rare Cajal cells. Hence any control tissue is bound to have a slightly deviating methylation profile due to its differentiation.

The patient’s genome harbours three heterozygous non-silent germline polymorphisms affecting the mitotic checkpoint proteins Aurora kinase A, BUB1 mitotic checkpoint kinase B, and succinate dehydrogenase subunit B, a germline polymorphism in the breast cancer 2 tumour suppressor and a polymorphism in the methylenetetrahydrofolate reductase protein.

The *SDHB* mutation (Arg90Gln) was to our knowledge only recently described in a single 1000Genomes project individual (dbSNP accession rs570278423). There are, however, three reported cases with a stopgain mutation in this codon in the context of paraglioma [42]. Codon 90 is part of the protein’s catalytic loop and is conserved in yeast and drosophila [43]. We can assert that this mutation did not disrupt protein translation, since immunostaining was positive. Speaking against a functional consequence of this mutation is the observation that SDH-deficient sporadic GIST that develop in adults display epithelioid morphology [44] which we did not observe in our sample. Furthermore, the literature considers that mutations in the SDH genes and in the KIT gene are mutually exclusive [45]. The *BUB1B* mutation (Thr40Met) was reported as a germline mutation in a case of colon cancer [46] and in a case of renal clear cell carcinoma [47] but has not been further validated as damaging. The *BRCA2* mutation (Asn372His) is weakly associated with an increased chance of breast cancer [48] but its incidence in some parts of the world is high. In two studies conducted in Italy [49] and Australia [50] the 372His variant was associated with increased risk of breast cancer. However, all three studies associate the homozygous variant to cancer risk, whereas in our case the mutation is heterozygous. The *AURKA* mutation (Phe31Ile) is determined to confer an increased risk for multiple types of cancer in several studies [51]. In our case the mutation is heterozygous. A study on gastric cancer patients in China [52] associated the homozygous *MTHFR* Ala222Val mutation with increased risk. In our patient the mutation is heterozygous. Although silent, the *RET* Ala45Ala allele (dbSNP ID rs1800858) was suggested to be either directly associated with or linked to another variant that conveys increased risk of Hirschsprung disease [53]. It was also found to be weakly correlated with multiple endocrine neoplasia type 2A [54].

A recent investigation [12] of 174 GIST cases identified three new canditate genes, *SYNE2*, *MAPK8IP2* and *DIAPH1*, displaying clinical relevance in the study. The study focused on expression level and copy number variation and is therefore not directly comparable to our analyses. The minimum we can say is that our sample harbours only known SNPs in these genes and only *SNYNE2* is affected by the 14q loss. Another recent paper [55] focuses on wild-type GIST and sets their gene expression pattern apart from those of kinase-mutant tumours. The complete datasets from these studies have not been published yet whence we can not draw further conclusions, e.g. about the expression of *RNF146* in GISTs.

The genetic and epigenetic analysis of a low-risk GIST presented here suggests that a typical activating *KIT* mutation together with loss of one of chromosomes 14 might indeed be sufficient to trigger formation of clinically symptomatic tumours. We can not, however, exclude the possibility that germline polymorphisms in the mitotic checkpoint genes *AURKA* and *BUB1B*, despite being heterozygous, facilitated tumour formation. In addition, we identified the uncharacterised zinc finger protein *ZNF407* as a potential GIST predisposition gene. A definite conclusion on the role of this protein can not be drawn without determining the target genes of ZNF407. This task we leave for future research.

## Supporting Information

S1 Table Clinical characteristics of the tumours included in the prevalence screen. Abbreviations: INA, information not available; AFIP, the Armed Forces Institute of Pathology. ^a^GIST patient in which the ZNF407 germline mutation was identified during prevalence screen; ^b^Patient’s tumor sample used for whole genome sequencing.
(HTML)
