# Supplementary material for: KIT Mutation and Loss of 14q May Be Sufficient for the Development of Clinically Symptomatic Very Low-Risk GIST
Source: PLoS One. 2015 Jun 23;10(6):e0130149. doi: 10.1371/journal.pone.0130149 (PMC4477893; doi:10.1371/journal.pone.0130149)
Supplement: S1 Table — Abbreviations: INA, information not available; AFIP, the Armed Forces Institute of Pathology. aGIST patient in which the ZNF407 germline mutation was identified during prevalence screen; bPatient’s tumor sample used for whole genome sequencing. (HTML) [file pone.0130149.s001.html]

Table

**Table S1. Clinical characteristics of the tumours included in the prevalence screen.**
Abbreviations: INA, information not available;
AFIP, the Armed Forces Institute of Pathology.
aGIST patient in which the ZNF407 germline mutation was identified during prevalence screen,
bPatient’s tumor sample used for whole genome sequencing.

| Case no. | Gender | Age | Tumor location | Histopathological subtype | AFIP/Fletcher risk | KIT/PDGFRA mutation |
| 1 | F | 78 | Stomach | Spindle | High | KIT 11 |
| 2 | M | 42 | Stomach | Mixed | Mod | PDGFRA 18 |
| 3 | M | 67 | Stomach | Mixed | High | KIT 11 |
| 4 | F | 81 | Stomach | Spindle | High | KIT 11 |
| 5 | F | 70 | Stomach | Spindle | Mod | KIT 11 |
| 6 | F | 73 | Stomach | Epithelioid | High | KIT 11 |
| 7 | F | 64 | Stomach | Spindle | Low | KIT 11 |
| 8 | M | 73 | Stomach | Spindle | High | KIT 11 |
| 9 | M | 52 | Stomach | Mixed | High | KIT 11 |
| 10 | M | 61 | Stomach | Spindle | Low | PDGFRA 18 |
| 11a | M | 62 | Stomach | Spindle | High | KIT 11 |
| 12 | F | 18 | Jejunum | Spindle | High | KIT 11 |
| 13 | M | 46 | Small intestine | Spindle | High | KIT 11 |
| 14 | F | 80 | Duodeno jejunal | Spindle | High | KIT 11 |
| 15 | M | 73 | Jejunum | Spindle | Mod | KIT 9 |
| 16 | M | 49 | Jejunum | Spindle | Mod | KIT 9 |
| 17 | M | 60 | Small intestine | Spindle | High | KIT 11 |
| 18 | F | 53 | Small intestine | Mixed | High | KIT 9 |
| 19 | F | 72 | Stomach | Spindle | High | KIT 11 |
| 20 | F | 68 | Stomach | Spindle | High | KIT 11 |
| 21 | F | 28 | Stomach | Spindle | High | KIT 11 |
| 22 | F | 57 | Stomach | Spindle | Low | KIT 11 |
| 23 | F | 67 | Jejunum | Mixed | Low | KIT 9 |
| 24 | M | 56 | Stomach | Epithelioid | Mod | PDGFRA 18 |
| 25 | F | 52 | Jejunum | Spindle | Low | KIT 11 |
| 26 | M | 62 | Stomach | Mixed | Mod | PDGFRA 18 |
| 27 | M | 54 | Stomach | Spindle | Mod | KIT 11 |
| 28 | F | 66 | Stomach | Spindle | Low | KIT 11 |
| 29 | M | 62 | Stomach | Spindle | Low | KIT 13 |
| 30 | F | 75 | Stomach | Spindle | Mod | KIT 11 |
| 31 | M | 58 | Stomach | Epithelioid | High | PDGFRA 18 |
| 32 | M | 92 | Stomach | Mixed | High | PDGFRA 18 |
| 33 | INA | 43 | Rectum | Spindle | Low | KIT 11 |
| 34 | INA | 73 | Stomach | INA | Very low | KIT 9 |
| 35b | M | 53 | Stomach | Spindle | Very low | KIT 11 |
| 36a | M | 80 | Esophagus | INA | Low | KIT 11 |
| 37 | INA | 72 | Stomach | Mixed | Very low | PDGFRA 18 |
| 38 | INA | 81 | Stomach | INA | Very low | KIT 11 |
| 39 | INA | 53 | Stomach | INA | Very low | KIT11 |
| 40 | INA | 60 | Duodenum | INA | High | KIT 9 |
| 41 | INA | 57 | Esophagus and proximal stomach | INA | High | KIT 11 |
| 42 | INA | 51 | Stomach | INA | High | KIT 11 |
| 43 | INA | 66 | Stomach | INA | Low | KIT 11 |
| 44 | INA | 75 | Stomach | INA | Very low | KIT 11 |
| 45 | INA | 75 | Stomach | INA | Very low | KIT 11 |
| 46 | INA | 67 | Stomach | INA | Very low | KIT 11 |
| 47 | INA | 71 | Stomach | INA | Low | PDGFRA 18 |
| 48 | INA | 63 | Duodenum | INA | Low | KIT 11 |
| 49 | INA | 50 | Small intestine | INA | Post Imatinib no grading | KIT 11 |
| 50 | INA | 70 | Small intestine | INA | High | KIT 11 |
| 51 | INA | 83 | Stomach | INA | High | KIT 11 |
| 52 | INA | 67 | Stomach | INA | Very low | KIT 11 |
